# Melatonin alleviates palmitic acid-induced mitochondrial dysfunction by reducing oxidative stress and enhancing autophagy in bovine endometrial epithelial cells

**DOI:** 10.1186/s40104-024-01064-x

**Published:** 2024-08-08

**Authors:** Yi Wang, Jianfei Gong, Nuo Heng, Yingfan Hu, Rui Wang, Huan Wang, Wei He, Ni Zhu, Zhihui Hu, Haisheng Hao, Huabin Zhu, Shanjiang Zhao

**Affiliations:** State Key Laboratory of Animal Biotech Breeding, State Key Laboratory of Animal Nutrition, Institute of Animal Science, Chinese Academy of Agricultural Sciences (CAAS), Beijing, China

**Keywords:** Autophagy, Bovine endometrial epithelial cells, Melatonin, Mitochondria, Oxidative stress, Palmitic acid

## Abstract

**Background:**

Negative energy balance (NEB) typically occurs in dairy cows after delivery. Cows with a high yield are more likely to experience significant NEB. This type of metabolic imbalance could cause ketosis, which is often accompanied by a decline in reproductive performance. However, the molecular mechanisms underlying NEB have yet to be fully elucidated. During excessive NEB, the body fat is extensively broken down, resulting in the abnormal accumulation of non-esterified fatty acids (NEFAs), represented by palmitic acid (PA), within the uterus. Such an abnormal accumulation has the potential to damage bovine endometrial epithelial cells (BEECs), while the molecular mechanisms underlying its involvement in the PA-induced injury of BEECs remains poorly understood. Melatonin (MT) is recognized for its regulatory role in maintaining the homeostasis of mitochondrial reactive oxygen species (mitoROS). However, little is known as to whether MT could ameliorate the damage incurred by BEECs in response to PA and the molecular mechanism involved.

**Results:**

Analysis showed that 0.2 mmol/L PA stress increased the level of cellular and mitochondrial oxidative stress, as indicated by increased reactive oxygen species (ROS) level. In addition, we observed mitochondrial dysfunction, including abnormal mitochondrial structure and respiratory function, along with a reduction in mitochondrial membrane potential and mitochondrial copy number, and the induction of apoptosis. Notably, we also observed the upregulation of autophagy proteins (PINK, Parkin, LC3B and Ubiquitin), however, the P62 protein was also increased. As we expected, 100 μmol/L of MT pre-treatment attenuated PA-induced mitochondrial ROS and restored mitochondrial respiratory function. Meanwhile, MT pretreatment reversed the upregulation of P62 induced by PA and activated the AMPK-mTOR-Beclin-1 pathway, contributing to an increase of autophagy and decline apoptosis.

**Conclusions:**

Our findings indicate that PA can induce mitochondrial dysfunction and enhance autophagy in BEECs. In addition, MT is proved to not only reduce mitochondrial oxidative stress but also facilitate the clearance of damaged mitochondria by upregulating autophagy pathways, thereby safeguarding the mitochondrial pool and promoting cellular viability. Our study provides a better understanding of the molecular mechanisms underlying the effect of an excess of NEB on the fertility outcomes of high yielding dairy cows.

## Introduction

Negative energy balance (NEB) is a common metabolic disorder in postpartum dairy cows. If NEB is not managed in a rapid and efficient manner, dairy cows, especially those with high milk production [1], will over-mobilize adipose tissue reserves to meet the needs of lactation, resulting in high concentrations of non-esterified fatty acids (NEFAs) in their blood. High concentrations of NEFAs, represented by palmitic acid (PA), has been proven to exert negative effects on the vitality and proliferation of bovine endometrial epithelial cells (BEECs), including the induction of apoptosis [2]. BEECs, as the first cells to engage in dialogue with the embryo, can determine whether an embryo can implant into the endometrium in early pregnancy. Consequently, the apoptosis of BEECs would inevitably influence the embryo implantation. A large number of studies have shown that the conception rate declines in cows with severe NEB, thus suggesting that an excess of NEFAs may affect the functionality of the endometrium, thereby affecting embryo implantation [3–5]. However, the mechanisms by which PA exerts effect on BEECs and embryo implantation have yet to be elucidated.

Palmitic acid is used as a substrate for fatty acid oxidation and is crucial for regulating energy homeostasis. As a driver of lipotoxicity, excessive PA leads to an increase in cellular reactive oxygen species (ROS), thus activating apoptosis in different cell types, including HepG2 cells [6], porcine intestinal epithelial cells [7] and HT-22 cells [8]. Significantly, myocytes, a type of cell that has high energy requirements, excessive PA can cause a reduction in mitochondrial membrane potential (MMP) [2], mitochondrial respiratory capacity [9, 10], and destroy mitochondrial DNA and morphology by reducing the expression of proteins involved in mitochondrial fusion, fission and biogenesis, leading to cell death. Thus, we hypothesized that PA could also induce mitochondrial dysfunction in BEECs, which in turn activates the apoptotic pathway.

When mitochondria are injured or become dysfunctional under oxidative excessive stress, the process of autophagy is initiated, acting as a self-clearance pathway that can remove oxidized cellular components and regulate the level of ROS in cells [11]. For example, in PA-treated hepatocytes, autophagy level was decreased, while adding the rapamycin, an mTOR inhibitor, to cell culture medium can effectively reverse the PA-induced repression of autophagy, and finally alleviate the aberrant lipid accumulation [12]. Similarly, Zhu et al. [13] reported that hepatic steatosis could be mitigated via activating AMPK/mTOR-mediated autophagy in aged mice fed a high-fat diet (HFD). However, the mechanism by which PA influence autophagy in BEECs and whether it is possible to rescue PA-treated BEECs by regulating autophagy remain uncertain.

Melatonin (MT), originally known as an effective antioxidant, is an endogenous hormone that is found throughout the body that involves in biological clock [14], circadian rhythm, reproductive physiology and others. Over recent decades, increasing attention to its unique function in regulating energy metabolism, especially in glucose and lipid metabolism, makes it a promising agent to open new avenues in the intervention of obesity. Injecting MT into HFD-fed mice could protect hepatocytes from lipotoxicity [15], demonstrated by a reduction in mitochondrial ROS as well as the mitigation of mitochondrial dysfunction. MT is also involved in autophagy to synergize with its antioxidant. In mice subjected to ischemia–reperfusion with MT pretreatment, the autophagic flux was enhanced while the level of apoptosis was reduced [16]. In the advent of neurotoxicity, MT prevented neuronal apoptosis by restoring mitochondrial autophagic activity through the AMPK-autophagy axis [17]. Additionally, exogenous MT treatment could promote the implantation potential of mouse blastocysts under oxidative stress induced by visible light [18, 19]. Collectively, these results illustrate that MT may play a dual role (antioxidant and autophagy) to protect cells, and may even protect individuals from harmful stimuli. Thus, there is a clear need to investigate whether MT can also regulate PA-induced autophagy in BEECs and identify its potential role in facilitating embryo implantation in cows with NEB.

In this innovative study, we investigated the effect of PA on mitochondrial homeostasis and autophagy in BEECs, and studied the mechanism by which MT-regulated autophagy and antioxidant effects could attenuate PA-induced damage in BEECs. Our findings provide new perspectives for the treatment of reproductive diseases in the cows with NEB.

## Materials and Methods

### Cell culture and treatment

BEECs, a stable cell line, were kindly gifted from Prof. Yong Guo’s team at the Beijing University of Agriculture [20], and revived into 100 mm culture dishes (430167, Corning, New York, USA) and subsequently passaged in various-sized culture dishes for various experiments. BEECs were cultured in DMEM/F12 (11330032, Gibco, California, USA) supplemented with 10% fetal bovine serum (FBS, 10091148, Gibco, California, USA) at 37 °C in a humidified atmosphere of 95% O_2_ and 5% CO_2_. Then, we selected BEECs in pebble shape with good adhesion for the experiments when they reached approximately 70% confluence. Based on the physiological serum levels of PA in NEB cows (approximately 0.133 to 0.4 mmol/L) [21, 22] and existing researches on PA [23–29], we exposed BEECs to 0.2 and 0.4 mmol/L of PA (KC002, Kunchuang biotechnology, Xi’an, China) for 12 and 24 h, respectively. Similarly, according to previous studies, we incubated BEECs with 100 μmol/L of melatonin (MT, M5250, Sigma, Missouri, USA) for 1 h before PA treatment to alleviate PA-induced damage in the BEECs [30–34].

### Immunofluorescence assays

BEECs were incubated in confocal dishes (D35-20-1-N, Cellvis, California, USA) at an initial seeding density of approximately 20,000 cells for 24 h. Cells were then fixed with 4% paraformaldehyde for 1 h at room temperature and then permeabilized with 0.5% Triton X-100 for 30 min. Next, the BEECs were blocked with phosphate-buffered saline (PBS, C10010500BT, Gibco, California, USA) containing 1% BSA at 4 °C overnight. The BEECs were then incubated with anti-CK18-specific antibody (10830-1-AP, Proteintech, Wuhan, China) overnight at 4 °C (with PBS as a negative control). The BEECs were then stained with fluorescein-coupled goat anti-rabbit IgG at 37 °C for 1 h. Finally, nuclei of BEECs were stained with DAPI (C1006, Beyotime, Shanghai, China) for 5 min and representative images were captured by confocal microscopy (TCS SP8, Leica, Wetzlar, Germany).

### Cell viability assays

Cell viability analysis was performed using a Cell Counting Kit-8 (C0037, Beyotime, Shanghai, China). BEECs were inoculated into 96-well plates (3599, Corning, New York, USA) and treated with different concentrations of PA. Treated cells were then incubated with 10 µL of CCK-8 solution at 37 °C for 1 h. Equal volumes of cell culture medium, PA and CCK-8 solution were used as blank controls. The optical density (OD) of each well was then measured at 450 nm using a microplate reader (Infinite 200 Pro, Tecan, Männedorf, Switzerland).

### EdU (5-ethynyl-2'-deoxyuridine) proliferation assay

EdU cell proliferation was assessed with a BeyoClick™ EdU-488 Cell Proliferation Assay Kit (C0071S, Beyotime, Shanghai, China). BEECs were inoculated into confocal dishes (D35-20-1-N, Cellvis, California, USA) and treated with 0.2 mmol/L and 0.4 mmol/L of PA for 12 h. EdU (10 μmol/L, 1 mL) reagent was then added to each well and incubated for 2 h to label the cells. Cells were subsequently fixed in 4% paraformaldehyde solution for 15 min at room temperature. Next, cells were then incubated with click reaction reagent for 30 min at room temperature in a dark environment. Nuclei were then re-stained using 1 × Hoechst 33342 reagent in the same environment. Representative images were finally captured by confocal microscopy (TCS SP8, Leica, Wetzlar, Germany).

### Apoptosis assays

Apoptosis was assessed using an Annexin V-FITC Apoptosis Detection Kit (C1062M, Beyotime, Shanghai, China). After treatment with PA or MT, the BEECs, which were cultured in 6-well plates (3516, Corning, New York, USA), were collected and resuspended with 195 µL of Annexin V-FITC conjugate. Then, we added 5 µL of Annexin V-FITC and incubated the cells at room temperature for 30 min. Finally, 5 µL of propidium iodide (PI) was added to stain the cells for 5 min. Unstained cells were used as a negative control, and FITC and PI single-stained cells were used as compensated controls. Next, the prepared cells were analyzed with a flow cytometer (FACSVerse, BD, New Jersey, USA). Analyses were performed using FlowJo software version V10.

### Oil Red O staining

An Oil Red O Staining Kit (G1262, Solarbio, Beijing, China) was used to stain BEECs, cultured in 6-well plates (3516, Corning, New York, USA), to observe the accumulation of intracellular lipids. Briefly, BEECs were first fixed with ORO fixative for 20 min. After washing with 60% isopropyl alcohol for 5 min, the cells were then stained with Oil Red O solution for 15 min at room temperature. The cells were then rinsed with distilled water to remove excess dye and the nuclei were re-stained with hematoxylin. Representative images were acquired by a light microscope (DM300, Leica, Wetzlar, German).

### RNA isolation and quantitative real-time polymerase chain reaction (qRT-PCR)

Total RNA was extracted from the BEECs, which were cultured in 6-well plates (3516, Corning, New York, USA), using a Total Cellular RNA Extraction Kit (DP430, TIANGEN, Beijing, China). Total RNA was then reverse-transcribed using a PrimeScript RT Kit with gDNA Eraser (6215A, Takara, Kyoto, Japan). Next, qRT-PCR assays were performed using the PowerUp™ SYBR™ Green Master Mix (A25742, ABI, Foster City, CA, USA) and QuantStudio™ 7 Flex System (ABI). The expression levels of target genes were then calculated using the 2^−ΔΔCT^ method. All primers were designed by the National Center for Biotechnology Information (NCBI). Primer information for each target gene is given in Table 1; the housekeeping gene β-actin was used as a reference.

**Table 1:** Nucleotide information

Primer name	GenBank accession No.	Sequence (5'→3')	PCR size, bp	T_m_, °C	Application
*β-Actin*-Forward	NM_173979.3	GCCCTGAGGCTCTCTTCCA	101	60	RT-PCR
*β-Actin*-Reverse		GCGGATGTCGACGTCACA			
*Caspase3*-Forward	NM_001077840	TACTTGGGAAGGTGTGAGAAAACTAA	71	59	RT-PCR
*Caspase3*-Reverse		AACCCGTCTCCCTTTATATTGCT			
*BCL2*-Forward	NM_001077486	GATGACTTCTCTCGGCGCTA	165	60	RT-PCR
*BCL2*-Reverse		GACCCCTCCGAACTCAAAGA			
*BAX*-Forward	NM_173894	GGCTGGACATTGGACTTCCTTC	112	61	RT-PCR
*BAX*-Reverse		TGGTCACTGTCTGCCATGTGG			
*PINK*-Forward	NM_001099701.2	GGAACTGGATGCAGATGGCT	261	60	RT-PCR
*PINK*-Reverse		CCCTGGCCGTAAAAGGGATT			
*PARKIN*-Forward	NM_001199065.1	AATCAAGAAGACCACCAAGCC	233	57	RT-PCR
*PARKIN*-Reverse		TGCGGTTCAGGAGGTTAAGAA			
*LC3B*-Forward	NM_001001169.1 [35]	TAAGGAAACCGTGCTGCTGT	124	60	RT-PCR
*LC3B*-Reverse		GCAGTGGTGTTTTTCCGTGT			
*PARL*-Forward	NM_001015596.1	TGGATAGCATAAGACCGCAGAA	186	52.3	RT-PCR
*PARL*-Reverse		AGGCTGGATTGGATGTGAAGTA			
*P62*-Forward	XM_024993877.1	CATTGCGGAGCCTCATCTCC	139	60	RT-PCR
*P62*-Reverse		CTCCGACACTCCTTCTTCTCTTT			

### Protein isolation and Western blotting

Total protein was extracted from BEECs that were cultured in 6-well plates (3516, Corning, New York, USA), using RIPA lysis buffer (IN-WB001, INVENT, Minnesota, USA) at 4 °C. Protein concentration was then determined with an Enhanced BCA Protein Assay Kit (P0010S, Beyotime, Shanghai, China). Protein samples were proportionally mixed with 5 × loading buffer (P06M18, Gene-Protein Link, Beijing, China) and boiled at 100 °C for 5 min. Next, up to 40 µg of proteins extracts were separated by SDS polyacrylamide gel electrophoresis (SDS-PAGE) and transferred onto a nitrocellulose membrane (HATF00010, Merck-Millipore, Darmstadt, Germany). The membranes were blocked with 5% skimmed milk for 2 h and then incubated overnight at 4 °C with primary antibody (Table 2). The membranes were then incubated with horseradish peroxidase-conjugated secondary antibody (Table 2) for 2 h at room temperature. Finally, the membranes were exposed with Chemistar^TM^ High-signal ECL Western blotting substrate (180–501, Tanon, Shanghai, China) and quantified by ImageJ software.

**Table 2:** Antibody information

Antibody name	Dilution ratio	Source	Cat. #
CK18	1:200	Proteintech	10830-1-AP
BAX	1:5,000	Proteintech	50599-2-Ig
BCL2	1:1,000	Proteintech	12789-1-AP
Caspase3	1:1,000	Proteintech	19677-1-AP
OXPHOS	1:1,000	Abcam	ab110413
P62	1:1,000	Proteintech	18420-1-AP
PINK	1:1,000	Cell Signaling Technology (CST)	2128S
PARKIN	1:5,000	Abcam	ab77925
LC3B	1:1,000	Proteintech	18725-1-AP
LAMP1	1:1,000	Proteintech	21997-1-AP
Cathepsin B (Cat-B)	1:1,000	Proteintech	12216-1-AP
Ubiquitin (Ub)	1:1,000	Proteintech	10201-2-AP
COX IV	1:1,000	Cell Signaling Technology (CST)	4850S
p-AMPK	1:1,000	Cell Signaling Technology (CST)	2535S
AMPK	1:1,000	Cell Signaling Technology (CST)	5831S
p-mTOR	1:1,000	Affinity	AF3308
mTOR	1:1,000	Affinity	AF6308
Beclin-1	1:1,000	Proteintech	11306-1-AP
β-Actin	1:1,000	Cell Signaling Technology (CST)	4970S
Goat anti rabbit IgG (H + L) -DyLight 488	1:1,000	Gene-Protein Link	P03S06S
Anti-rabbit IgG HRP-linked antibody	1:2,000	Cell Signaling Technology (CST)	7074S

### Assays to determine the levels of reactive oxygen species (ROS) in cells and mitochondria

Levels of cellular ROS (cROS) were detected with a ROS assay kit (S0033S, Beyotime, Shanghai, China) in BEECs that were cultured in 6-well plates (3516, Corning, New York, USA). The treated cells were collected and suspended in 10 µmol/L of DCFH-DA and incubated at 37 °C for 30 min. Samples were inverted every 10 min. Next the cells were washed three times with DMEM/F12. Finally stained cells were analyzed with a flow cytometer (FACSVerse, BD, New Jersey, USA). Analyses were performed using FlowJo software version V10.

The MitoSOX™ Red Mitochondrial Superoxide Indicator (M36008, Invitrogen, California, USA) was used to detect ROS levels in mitochondria. Treated BEECs, which were cultured in 6-well plates (3516, Corning, New York, USA), were collected and stained with 500 nmol/L of MitoSOX™ and incubated for 30 min at 37 °C in the dark. The mixture was inverted every 10 min. Then, the BEECs were washed three times in DMEM/F12. Finally, the stained cells were analyzed by using a flow cytometer (FACSVerse, BD, New Jersey, USA). Analyses were performed using FlowJo software version V10.

### Mitochondrial membrane potential (MMP) assay

Next, we used the JC-1 probe (C2006, Beyotime, Shanghai, China) to detect the MMP. Flow cytometry and immunostaining were used to demonstrate changes in MMP. Treated cells, which were cultured in 6-well plates (3516, Corning, New York, USA), were collected and incubated with 0.5 mL of JC-1 working solution (1×) at 37 °C for 20 min, mixing once every 5 min. Cells were then washed twice with JC-1 staining buffer (1×). Finally, the cells were analyzed with a flow cytometer (FACSVerse, BD, New Jersey, USA). Analyses were performed using FlowJo software version V10. Unlike the flow cytometer method, staining was performed directly in a confocal petri dish without collecting cells. Representative images were captured by confocal microscopy (TCS SP8, Leica, Wetzlar, Germany) for analysis.

### MDA, SOD assays

Malondialdehyde (MDA) levels were assessed with the Lipid Peroxidation MDA Assay Kit (S0131S, Beyotime, Shanghai, China). In brief, PA-treated BEECs, which were cultured in 6-well plates (3516, Corning, New York, USA), were lysed with RIPA; then, protein concentrations were determined with a BCA kit. Next, 100 µL of cell lysate was mixed with 200 µL of MDA working solution for 15 min at 100 °C. After centrifugation at 1,000 × *g* for 10 min, 200 µL of supernatant was transferred to 96-well plates (3599, Corning, New York, USA). A microplate reader was then used to detect the absorbance at 532 nm. The final intracellular MDA level per protein weight was then calculated from the protein concentration.

Cellular SOD activity was determined with a CuZn/Mn-SOD Activity Assay Kit (WST-8 method) (S0103, Beyotime, Shanghai, China) in BEECs that were cultured in 6-well plates (3516, Corning, New York, USA). In brief, cell homogenates were generated with an ultrasonic cell crusher and protein concentrations were determined with a BCA kit. Next, a mixture of 20 µL of treated sample, 160 µL of WST-8 test solution, and 20 µL of reaction starter was placed in a 96-well plate (3599, Corning, New York, USA) and incubated for 30 min at 37 °C. Absorbance readings were taken at 450 nm using a microplate reader. The final intracellular SOD activity per protein weight was calculated from the protein concentration.

### Transmission *electron* microscopy (TEM)

For TEM, cells were seeded on ACLAR^®^ 33C Film (#50425, Electron Microscopy Sciences, Pennsylvania, USA) and fixed with 2.5% glutaraldehyde (#G5882, Sigma, Missouri, USA) in 0.1 mol/L PB buffer (pH 7.4). Samples were thoroughly washed in 0.1 mol/L PB buffer (pH 7.4), and osmicated with 1% OsO_4_ and 0.8% potassium ferrocyanide (#60279, Sigma) for post-fixation for 30 min at room temperature in the dark. Next, samples were incubated in 1% aqueous uranyl acetate, rinsed in distilled water and dehydrated in a series of ethanol solutions (30%, 50%, 70%, 85%, 95% and 100%; 6 min each) and dehydrated with acetone (2 × 100%, 6 min/incubation). Subsequently, cells were gradually equilibrated with EMbed 812 resin (#14120, Electron Microscopy Sciences) and then solidified at 65 ℃ for 24 h. Next, the ACLAR 33C film were removed and resin blocks were trimmed. Then, ultra-thin sections (70 nm) were created with an ultramicrotome (Leica Microsystem UC7) equipped with a diamond knife (ultra 35°, Diatome, Switzerland). Serial sections were collected on copper grids with a single slot and counterstained with uranyl acetate and lead citrate. The grids were then viewed on a transmission electron microscope (Tecnai G^2^ Spirit BioTWIN, Thermo Fisher Scientific, MA, USA) operating at 120 kV. Images were acquired with a digital camera (Orius 832, Gatan, California, USA) for analysis.

### Transfection with Ad-mCherry-GFP-LC3B

We infected cells at 50% confluence in a confocal dish (D35-20-1-N, Cellvis, California, USA) with 20 MOI Ad-mCherry-GFP-LC3B (C3011, Beyotime, Shanghai, China) for 24 h and then treated the cells with PA for 12 h. Following fixation and permeabilization, the cells were stained with DAPI staining solution for 3 min in the dark. Finally, the stained cells were observed by confocal microscopy (TCS SP8, Leica, Wetzlar, Germany).

### Extraction of mitochondria

We extracted mitochondria from BEECs that were cultured in 100 mm culture dishes (430167, Corning, New York, USA) using the Minute™ Mitochondria Isolation Kit for Muscle Tissues/Cultured Muscle Cells (MM-038, INVENT, USA) for subsequent Western blotting experiments. The cells were resuspended by adding 50 μL of buffer A and transferred to a centrifuge column cannula. Next, 80 mg of tissue grinding powder was added to the centrifuge column and ground for 3 min with repeated downward pressure and twisting with a plastic rod. Then, we added 300 μL of buffer A to the column, followed by centrifugation at 16,000 × *g* for 30 s. The centrifuge column was then discarded and the precipitate was vortexed in the receiver tube and centrifuged at 1,000 × *g* for 5 min. Then, the supernatant was transferred to a new 1.5-mL centrifuge tube and centrifuged at 11,000 × *g* for 20 min. Next, 200 μL of buffer B was added to the precipitate which was resuspended by blowing 30 times, and vortexed vigorously for 20 s. Next, the samples were centrifuged for 10 min at 11,000 × *g*, and the supernatant was transferred to a new 1.5-mL centrifuge tube, mixed with 0.3 mL of pre-cooled PBS, and mixed thoroughly by vortexing. Next, the samples were centrifuged for 20 min at 16,000 × *g*; the resultant precipitate contained the mitochondria. Mitochondrial proteins were subsequently extracted by adding RIPA lysate to the precipitate.

### LysoTracker and LysoSensor staining assays

The treated BEECs, cultured in confocal dishes (D35-20-1-N, Cellvis, California, USA), were incubated with 50 nmol/L Lyso-Tracker Red (C1046, Beyotime, Shanghai, China) at 37 °C for 30 min and then incubated with 1 × Hoest (C0071S, Beyotime, Shanghai, China) at 37 °C for 10 min. Finally, the stained cells were observed under a confocal microscope (TCS SP8, Leica, Wetzlar, Germany).

The treated BEECs, cultured in confocal dishes (D35-20-1-N, Cellvis, California, USA), were incubated with 5 µmol/L LysoSensor™ Yellow/Blue DND-160 (L7545, Invitrogen, California, USA) at 37 °C for 1 h and incubated with 1 × Hoest (C0071S, Beyotime, Shanghai, China) at 37 °C for 10 min. Finally, the stained cells were observed under a confocal microscope (TCS SP8, Leica, Wetzlar, Germany).

### Mitochondrial respiration

Oxygen consumption rate (OCR) was determined using a Flux XF-96 analyzer (XFe96, Seahorse Bioscience, Massachusetts, USA). Cells were seeded at a density of 1 × 10^4^ per well in XF 96-well cell culture microplates (Seahorse Bioscience) and incubated overnight at 37 °C and 5% CO_2_. Then, PA was added to the BEECs for 12 h. Prior to mounting, basal medium (102353-100, Seahorse Bioscience) containing 10 mmol/L of glucose (G7528, Sigma), 1 mmol/L of sodium pyruvate (S8636, Sigma) and 2 mmol/L of glutamine (G8540, Sigma) were added and cells were incubated at 37 °C for 1 h under CO_2_-free conditions. The following reagents were sequentially added to the corresponding dosing wells: 1 μmol/L of oligomycin (ab141829, Abcam, Cambridge, UK), 1 μmol/L of Fccp (C2920, Sigma), and a mixture of 1 μmol/L of rotenone (R8857, Sigma) and 1 μmol/L of antimycin A (ab141904, Abcam). When calculating OCR, at least three wells per condition were used; these were normalized by cell number.

### DNA extraction and mitochondrial copy number detection

DNA was extracted from BEECs that were cultured in 6-well plates (3599, Corning, New York, USA) using the TIANamp Genomic DNA Kit (DP304, TIANGEN, Beijing, China). Buffer GA, proteinase K, and buffer GB, were added to the collected cells which were then incubated at 70 °C for 10 min. Further 200 µL of anhydrous ethanol was added and the sample was shaken well for 15 s. These solutions were added to an adsorbent column and centrifuged at 12,000 r/min for 30 s; excess solution was poured off. Then, buffer GD, and rinse solution PW, were added to remove impurities. Finally, TE elution buffer was added to the adsorption column and the DNA solution was centrifuged at 12,000 r/min for 2 min. Mitochondrial copy number assay was performed by qRT-PCR, using DNA as the substrate. Three fragments located on mitochondrial DNA were selected as mitochondrial target genes (*COX1, ND1* and *ATP6)* and the stable cytosolic single-copy gene *AGRT1* on *Bos taurus* was chosen as a reference gene. Primer information is given in Table 3.

**Table 3:** mtDNA nucleotide information

Primer name	GenBank accession No.	Sequence (5'→3')	Application
*COX1*-Forward	NC_006853.1(5687..7231)	CCTCTATAGTTGAAGCTG	mtDNA
*COX1*-Reverse		TTGTAATGAAGTTGATGG	
*ND1*-Forward	NC_006853.1(3101..4056)	ACTAATTATTCCCATCCTATTGGCC	mtDNA
*ND1*-Reverse		AAGATGTAGCGGGTCGTAGTGGTTC	
*ATP6*-Forward	NC_006853.1(8290..8970)	TCCCAACATCAAACCGACTA	mtDNA
*ATP6*-Reverse		AATTACGGCTCCTGCTCACA	
*AGRT1*-Forward	NC_037328.1	TGCCAGCGTGTTTCTACT	mtDNA
*AGRT1*-Reverse		AAGCCTTCTTGAGGGTCT	

### Statistical analysis

All experiments involved at least three independent biological replicates and all data are expressed as mean ± SEM. Statistical analyses were performed using SAS software version 9.2. Comparisons between two groups were performed two-tailed unpaired Student’s *t*-test. One-way analysis of variance (ANOVA) was also used for multiple comparisons. *P*-values ≤ 0.05 were considered statistically significant.

## Results

### PA caused apoptosis in BEECs

**Fig. 1:**
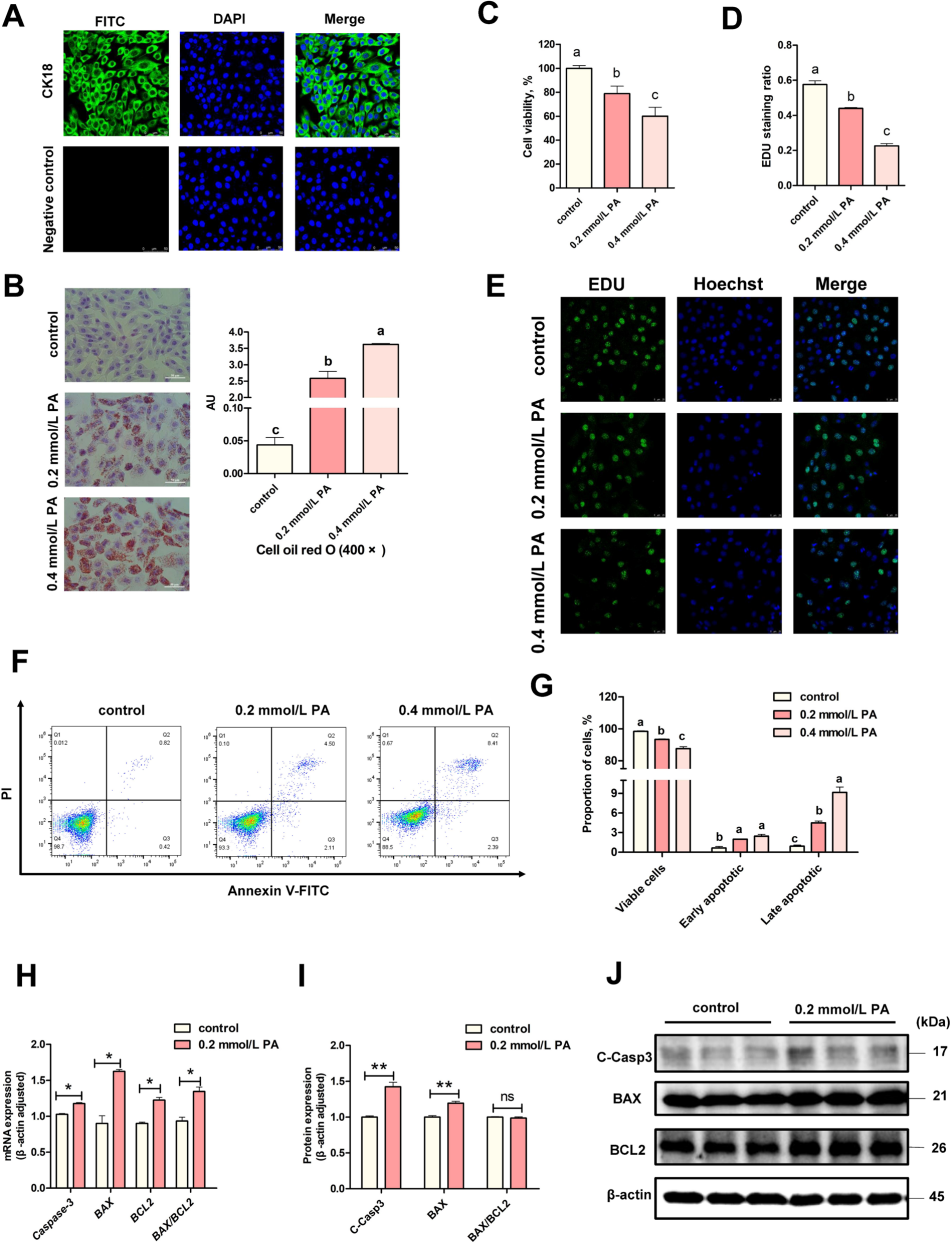
PA caused apoptosis in BEECs. **A** Representative images of CK18 staining in BEECs (scale bars = 50 μm). **B** Representative images and analysis of Oil Red O staining (× 400) (*n* = 3). **C** Cell viability of BEECs under different concentrations of PA stimulation (*n* = 5). **D** Representative images of EDU staining. **E** Analysis of EDU staining (*n* = 3). **F** and **G** Apoptosis of BEECs in the control and PA treated groups were analyzed by flow cytometry after the stimulation of BEECs with PA (*n* = 3). **H** qRT-PCR analysis of *BAX, BCL2, Caspase3*, in the control and PA groups (*n* = 3). **I** and **J** Western blot analysis of BAX, BCL2, cleaved-caspase3, in the control and PA groups (*n* = 3). For B–D, H and I, we used one-way ANOVA for significant difference analysis (different lower-cases letters indicate *P* < 0.05); for H and I, we used Student’s* t*-test to identify significant differences (^*^P < 0.05;^*^P < 0.01); Date are shown as mean ± SEM

Following recovery and culture, the cells were observed to be in good condition. According to immunofluorescence (IF), more than 99% of cells expressed the epithelial cell marker keratin CK18 (Fig. 1A), indicating that the cells were pure and could be used for subsequent experiments. We constructed a high-fat stress model with PA; the lipid content of BEECs increased significantly in a dose dependent manner following PA treatment (Fig. 1B, *P* < 0.05). These findings proved that the model had been established successfully. Next, we found that both cell viability (Fig. 1C, *P* < 0.05) and proliferation rate (Fig. 1D and E, *P* < 0.05) were notably decreased in a dose-dependent manner. Flow cytometry data also showed a significant reduction in the number of surviving cells following 0.2 mmol/L of PA (*P* < 0.05); and the proportion of early apoptotic and late apoptotic cells was significantly higher compared to the control group (Fig. 1F and G, *P* < 0.05). Based on the cell viability, proliferation and apoptosis data, we chose 0.2 mmol/L of PA treatment for subsequent experiments. Next, we detected apoptosis-related mRNA and protein expression after 0.2 mmol/L of PA treatment. The results showed that the mRNA expression of pro-apoptotic genes (*Caspase3, BAX/BCL2*) were significantly elevated (*P* < 0.05) and apoptosis-associated proteins (cleaved-Caspase3, BAX) were also significantly up-regulated after PA treatment (Fig. 1H–J, *P* < 0.01). However, there was no difference in the expression levels of BAX/BCL2 proteins. Collectively, these data showed that PA treatment induced apoptosis in BEECs.

### PA enhanced peroxidation in BEECs

**Fig. 2:**
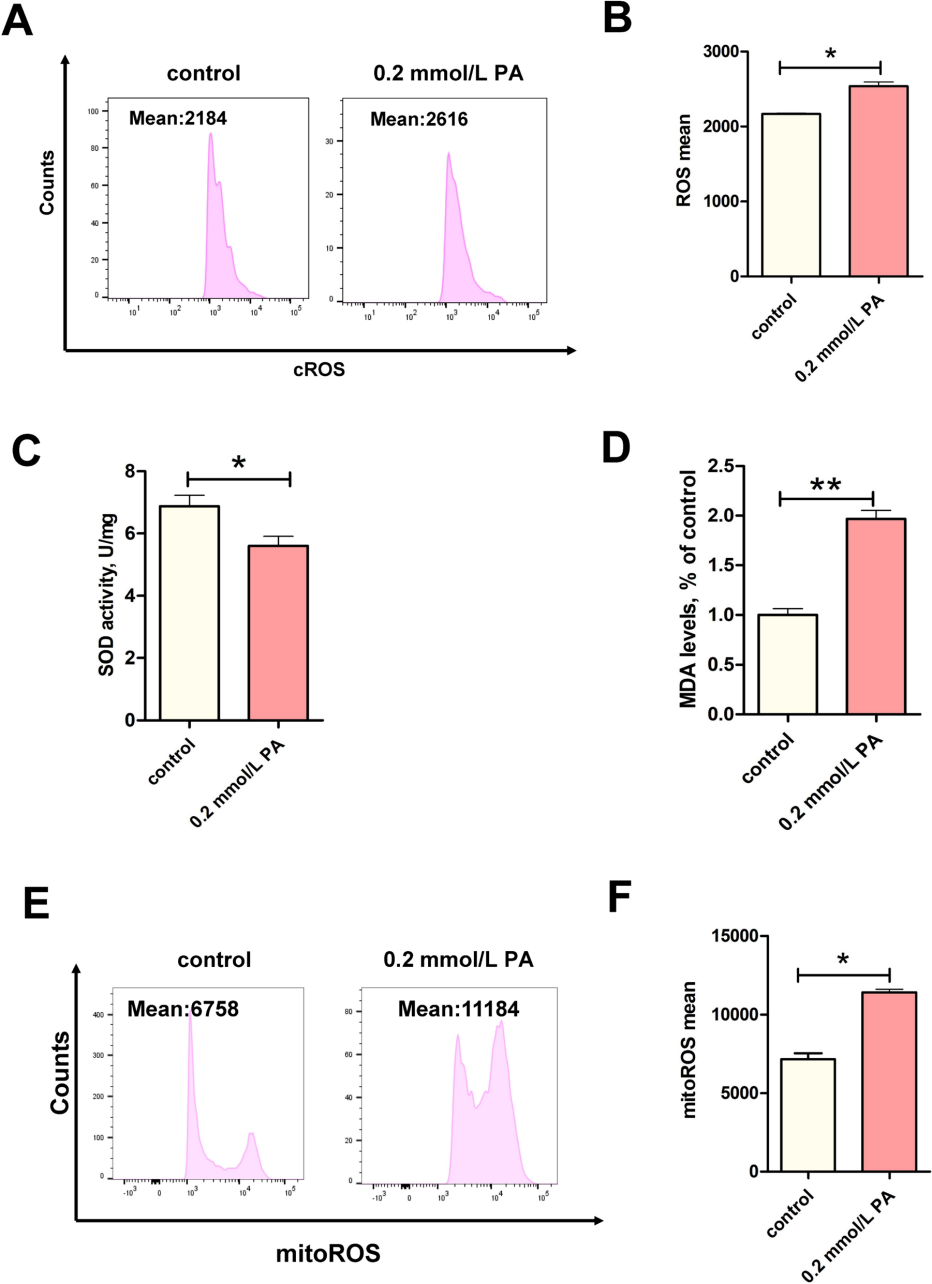
PA induced mitochondrial oxidative stress in BEECs. **A** and **B** cROS levels in the control and PA-0.2 mmol/L groups (*n* = 3). **C** MDA levels in the control and PA-0.2 mmol/L groups (*n* = 3). **D** SOD activity in the control and PA-0.2 mmol/L groups (*n* = 5). **E** and **F** MitoROS levels in the control and PA-0.2 mmol/L groups (*n* = 3). For B**–**D and F, we used Student *t*-test identify significant differences (^*^P < 0.05; ^**^P < 0.01); Date are shown as mean ± SEM

Apoptosis induced by PA-generated lipotoxicity is often associated with oxidative stress. We found the levels of both intracellular and mitochondrial ROS were significantly upregulated (Fig. 2A–B and 2E–F, *P* < 0.05). In addition, levels of the oxidative product MDA increased significantly under PA stimulation (Fig. 2C, *P* < 0.01), and the activity of antioxidant enzyme SOD decreased significantly (Fig. 2D, *P* < 0.05). Our data suggest that the PA-induced increase in apoptosis in BEECs may be related to oxidative damage, especially in mitochondria.

### PA induced mitochondrial dysfunction in BEECs

**Fig. 3:**
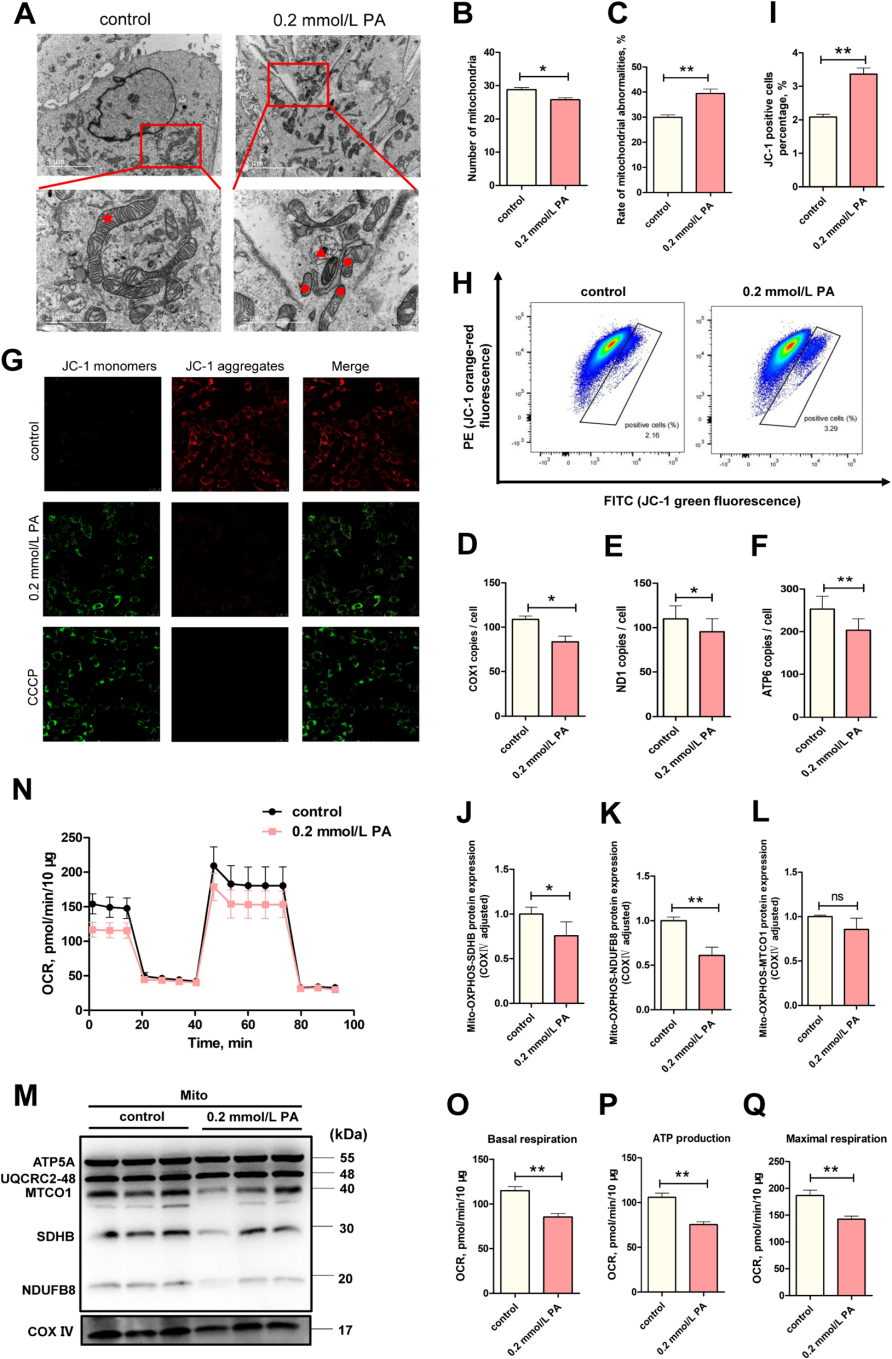
PA contributed to mitochondrial dysfunction in BEECs. **A** Representative images of TEM (scale bars = 5 and 2 μm) in the 3 groups. Normal mitochondria are marked with red stars, abnormal mitochondria are marked with red circles, and lysosomes are marked with red triangles. **B** TEM analysis of mean mitochondrial number (*n* = 3). **C** TEM analysis of abnormal mitochondria (*n* = 3). **D**–**F** Mitochondrial copy number analyses was determined by specific *COX1, ND1*, and *ATP6* gene assays (*n* = 3). **G** Representative images of JC-1 staining in the control and PA-0.2 mmol/L groups (*n* = 5). **H** and **I** Representative images and analysis plots of JC-1 cells as determined by flowcytometry (*n* = 3). **J**–**M** Western blot analysis of OXPHOS in the control and PA groups (*n* = 3). **N** OCR in the control and PA-0.2 mmol/L groups. **O–Q** Basal respiratory and ATP production and maximal respiration, as determined by the Seahorse analyzer (*n* = 4). We used Student’s *t*-test to identify significant differences (^*^P < 0.05; ^**^P < 0.01); Date are shown as mean ± SEM

PA is an energy metabolism substrate, and mitochondria, as organelles that perform fatty acid oxidation in cells, may suffer damage to their structure and function in BEECs with elevated levels of mitochondrial ROS after PA stress. TEM demonstrated that mitochondria in the PA group underwent swelling, lysis of the cristae, and membrane rupture (Fig. 3A). Statistical analysis revealed a significant increase in the proportion of damaged mitochondria compared to the control group (Fig. 3C,* P *< 0.05). Damage to mitochondrial structure often leads to mitochondrial dysfunction, such as a decrease in MMP, as demonstrated by JC-1 staining (Fig. 3G) and cell flow cytometry (Fig. 3H and I). Extracted mitochondria incubated with OXPHOS antibodies showed a significantly reduced expression levels of NDUFB8 (Complex I,* P* < 0.05) and SDHB (Complex II, *P* < 0.01) proteins, and a downward trend in MTCO1 (Complex IV) (Fig. 3J–M), indicating that PA impairs functionality in the electron respiratory chain. OCR analysis with the Seahorse analyzer, demonstrated that basal cellular respiration, ATP production, and maximal oxygen consumption all diminished following PA stimulation (Fig. 3N–Q, *P* < 0.01), which are the consequences of an impaired mitochondrial respiratory chain. These observations indicated that PA-induced mitochondrial dysfunction helps to drive injury in BEECs. Interestingly, we found the number of short rod-shaped mitochondria increased, which may be divided from mitochondria as the damaged part. Furthermore, we identified a reduction in mitochondrial number by TEM (Fig. 3B, *P* < 0.05), along with a reduction of mtDNA levels (Fig. 3D–F), which was likely attributed to active autophagy.

### PA activated autophagy in BEECs

**Fig. 4:**
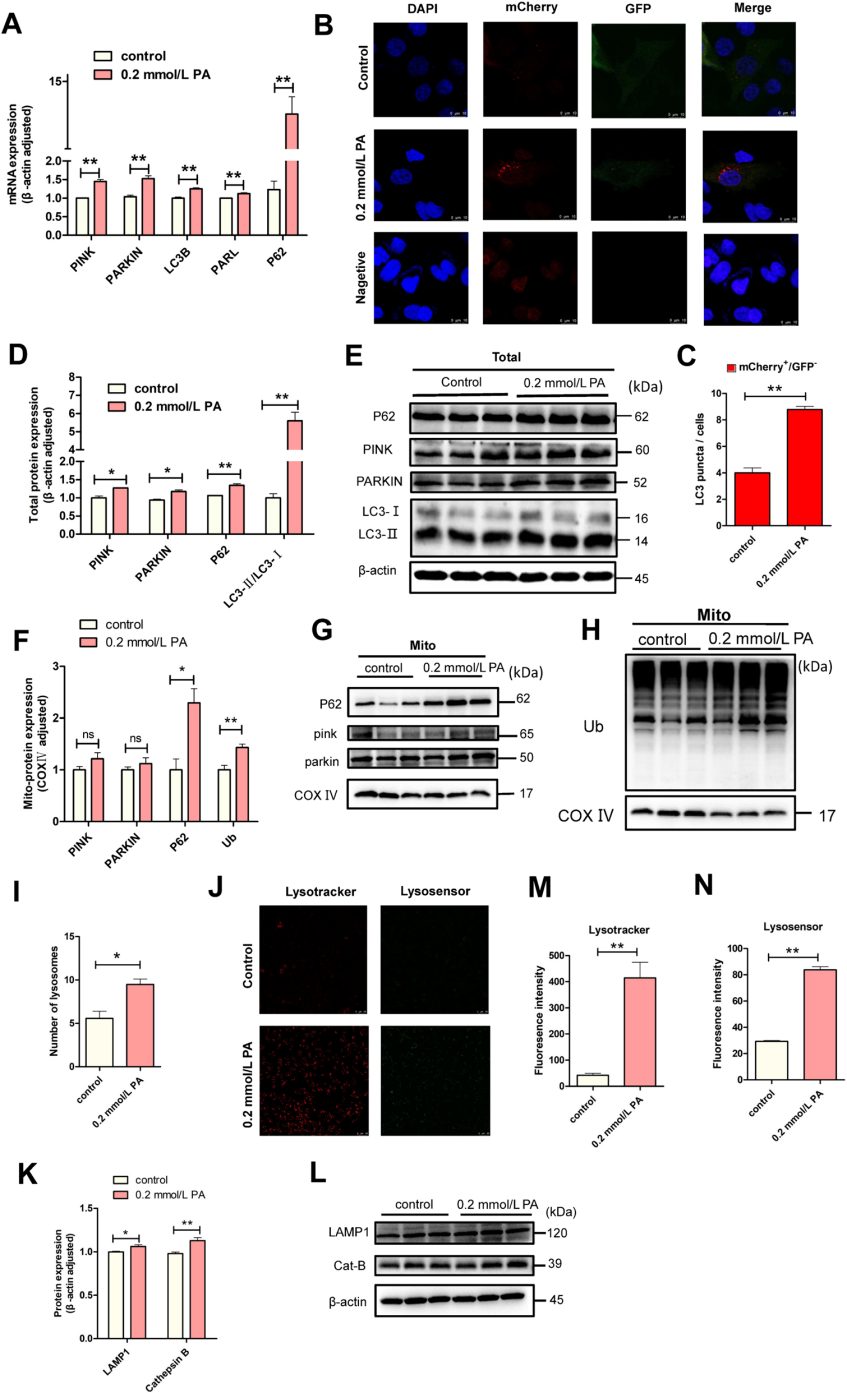
PA enhanced mitochondrial autophagy in BEECs. **A** qRT-PCR analysis of *PINK*, *PARKIN*, *LC3B*, *PARL*, *P62* in the control and PA groups (*n* = 3). **B** and **C** Representative confocal images and statistical analysis following the transfection of mcherry-GFP-LC3B in the control and PA groups (*n* = 3). **D** and **E** Western blot analysis of PINK, PARKIN, LC3B and P62 protein in control and PA groups (total protein, *n* = 3). **F**–**H** Western blot analysis of PINK, PARKIN, P62 and Ub in the mitochondria from control and PA groups (*n* = 3). **I** Quantitative analysis of lysosomes, as determined by TEM (*n* = 3). **J** Representative confocal images of LysoTracker and LysoSensor in the control and PA groups (scale bars = 50 μm). **K** and **L** Western blot analysis of LAMP1 and Cat B in control and PA groups (total protein, *n* = 3). **M** Fluorescence intensity analysis of LysoTracker staining (*n* = 4). **N** Fluorescence intensity analysis of LysoSensor staining (*n* = 4). We used Student’s *t*-tests to identify significant differences (^*^P < 0.05; ^**^P < 0.01); Date are shown as mean ± SEM

To investigate whether PA could activate autophagy, we first detected the mRNA expression of genes related to mitochondrial autophagy (*PINK*, *PARKIN*, *LC3B*, *PARL*, and *P62*), and found they all significantly increased after treatment with 0.2 mmol/L of PA (Fig. 4A, *P* < 0.01). Meanwhile, the protein expression levels of PINK, PARKIN, LC3-II/I and P62 in BEECs also increased (Fig. 4D and E), thus revealing that more mitochondria were marked to autophagy. We further extracted mitochondria to explore the expression of PINK, PARKIN, P62 and ubiquitin (Ub), which are located or connected to mitochondria during pre-autophagy. The levels of P62 and Ub proteins were significantly increased, while the levels of PINK and PARKIN were up-regulated (but not significantly, *P* > 0.05), further supporting our previous results (Fig. 4F–H). Then, mCherry-GFP-LC3B transfection revealed a significant increase in the red spots in the BEECs following PA treatment (Fig. 4B and C, *P *< 0.01), which preliminarily suggested that more autophagosomes have formed. The pre-autophagy was well prepared proved by above results. Furthermore, the number of lysosomes in BEECs was significantly increased after PA treatment, as determined by TEM (Fig. 4I, *P* < 0.05). LysoTracker staining also indicated that the number of lysosomes increased significantly (Fig. 4J and M). Besides the increased number of lysosomes, the LysoSensor staining with stronger fluorescence intensity in PA group showed that PA also reduced pH value significantly (Fig. 4J and N). The increased protein expression of LAMP1 and Cat B were consistent with our earlier findings (Fig. 4K and L). It is possible that the cells increased both lysosome number and function to match the increased demand of the larger number of autophagosomes. In view of the pre- and post-autophagy stages, mitochondrial autophagy was really enhanced after PA treatment. However, it is also important to note that the expression levels of P62 protein were significantly elevated (Fig. 4D, *P* < 0.01; Fig. 4F, *P* < 0.05); this may be the main reason why autophagy was enhanced but failed to prevent apoptosis.

### MT reduced the PA-induced apoptosis in BEECs by decreasing ROS production

**Fig. 5:**
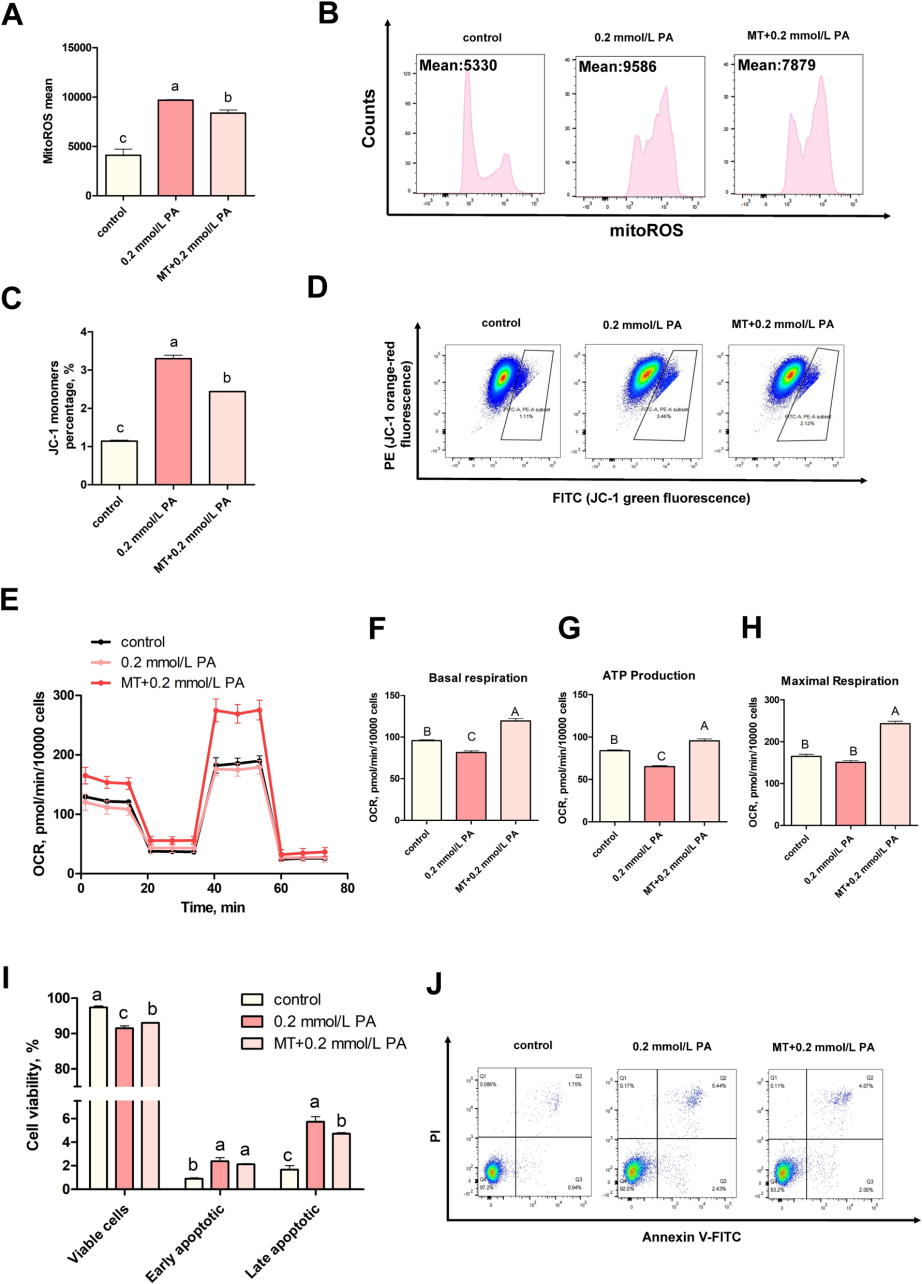
MT reduced the PA-induced apoptosis by reducing ROS production in BEECs. **A** and **B** MitoROS levels in the control, PA-0.2 mmol/L and MT groups (*n* = 3). **C** and **D** Representative images and analysis plots of JC-1, as determined by flow cytometry (*n* = 3). **E** OCR levels in the control, PA-0.2 mmol/L and MT groups. **F**–**H** Basal respiratory and ATP production, and maximal respiration, as determined by the Seahorse analyser (*n* = 4). **I** and **J** The extent of apoptosis in BEECs was analyzed by flow cytometry. For A, C and F–I, we used one-way ANOVA to identify significant differences (the different lower-cases numbers represent *P* < 0.05 and different upper cases letters represent *P* < 0.01); Date are shown as mean ± SEM

MT performs a dual role as both an antioxidant and a regulator of autophagy. We incubated BEECs with 100 μmol/L of MT for 1 h before adding PA. Flow cytometry showed that MT treatment significantly reduced the mitoROS production induced by PA (Fig. 5A and B, *P* < 0.05). MT pretreatment further attenuated PA-induced mitochondrial dysfunction. We found that MT increased the mitochondrial membrane potential (Fig. 5C and D, *P* < 0.05). In addition, we found that MT pretreatment significantly increased basal respiration, ATP production, and maximal respiration, when compared with the PA-treated group, as determined by the Seahorse analyser, suggesting that MT pretreatment enhances mitochondrial respiratory function following PA treatment (Fig. 5E–H, *P* < 0.01). Finally, the proportion of viable cells and the proportion of late apoptotic cells in BEECs from the MT pretreatment group were significantly higher than those in the PA-treated group (Fig. 5I and J, *P *< 0.05), suggesting that MT contributed to the reduction of PA-induced apoptosis.

### MT further enhanced PA-induced autophagy

**Fig. 6:**
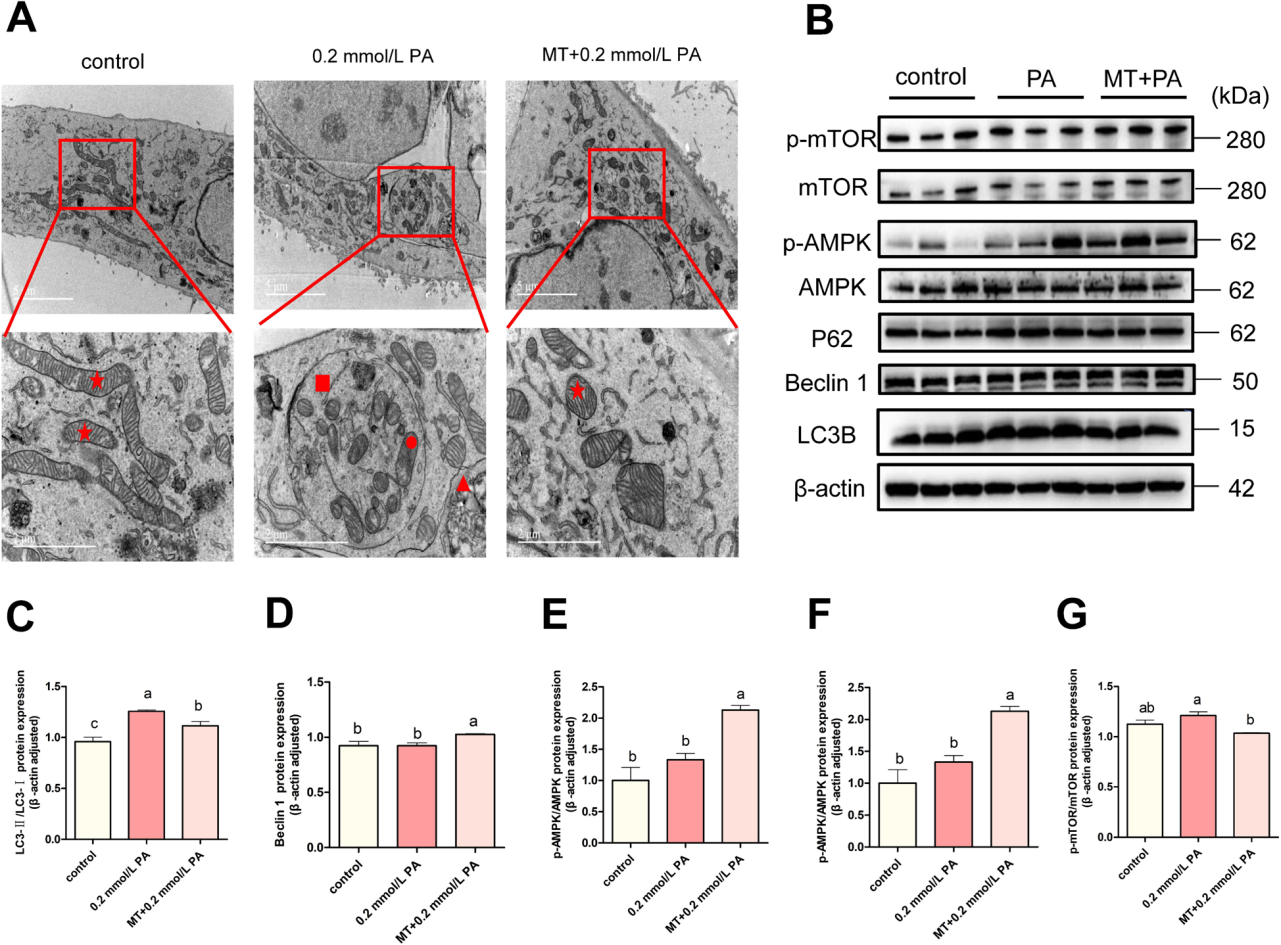
MT further enhanced PA-induced autophagy. **A** Representative TEM images (scale bars = 5 and 2 μm) acquired from the three groups. Normal mitochondria are marked with red stars, abnormal mitochondria are marked with red circles, lysosomes are marked with red triangles, and autolysosomes are marked with red squares (*n* = 3). **B**–**G** Western blot analysis of p-AMPK/AMPK, p-mTOR/mTOR, Beclin1, LC3II/I and P62 proteins (*n* = 3). We used one-way ANOVA to identify significant differences (different lower cases letters represent *P* < 0.05); Date are shown as mean ± SEM

Compared with the PA-treated group, fewer number of lysosomes were detected following MT pretreatment, consistent with the results of further increased level of autophagy in the MT group (Fig. 6A). Further, compared with PA group, the expression levels of p-AMPK/AMPK and Beclin1 proteins were significantly higher, whereas the expression levels of p-mTOR/mTOR, LC3BII/I and P62 proteins were significantly lower (Fig. 6B–G, *P* < 0.05), suggesting that MT could further activate autophagy via the AMPK/mTOR/Becline-1 pathway. In conclusion, MT pretreatment alleviated PA-induced cellular damage by reducing oxidative stress and further enhancing autophagy.

## Discussion

Here, we presented evidence that accumulation of PA induced mitochondrial oxidative stress and dysfunction, while modulating the level of mitochondrial autophagy in BEECs. Moreover, pre-treatment MT protected cells by attenuating mitochondrial oxidative stress and by enhancing autophagy through the AMPK-mTOR-Beclin1 pathway.

PA, as the most abundant saturated fatty acid present in the circulation, has the capacity to initiate oxidative stress in a variety of cell types, subsequently precipitating cellular apoptosis [36–39]. Meanwhile, it is known to be associated with recurrent pregnancy loss during human assisted reproductive technology [40, 41]. Thus, our study specially focused on the effects of PA on the endometrium by applying a PA-treated BEECs model. Consistent with previous studies, we found that PA stimulation induced excessive apoptosis of cells [42–44]. Also, PA caused oxidative stress in BEECs, as demonstrated by the significantly increase in intracellular and mitochondrial ROS levels, a higher MDA content, and a reduction in SOD activity. By combining the swelling and cristae rupture of mitochondria, we confirmed that the oxidative stress induced by PA may damage the mitochondria [45].

Moreover, our results showed that Complex I and II proteins in the electron transport chain (ETC) were significantly decreased, and Complex III protein was on a downward trend, which revealed the ETC was disturbed by PA. Complexes I, III and IV, as proton pumps in the ETC, are responsible for generating the MMP [46, 47]. Morganti et al. [48] revealed that, the Complex II was inhibited when the MMP in hematopoietic stem/progenitor cells was attenuated. As expected, the reduction in Complex I, II and III protein was accompanied by a decrease in MMP and ATP production. Further, the slower energy metabolism and oxygen consumption detected by the Seahorse analyzer identified this hypothesis, PA would damage the ETC, which in turn induced mitochondrial dysfunction. Consistent with our hypothesis, both York et al. [49] and MacDermott-Opeskin et al. [50] have discovered that fatty acids could act as uncoupling agents for oxidative phosphorylation via deprotonated sites, leading to the breakdown of mitochondrial proton gradients and the release of oxidative phosphorylation.

In addition to oxidative stress and mitochondrial dysfunction, TEM analysis also revealed a decreased number of mitochondria under PA treatment. Where did they go? It is possible that BEECs cannot endure an unhealthy environment alongside damaged mitochondria and initiate autophagy to remove damaged mitochondria, leading to a reduction in stimulation. Moreover, our data showed an improvement of autophagy in PA-treated BEECs, as demonstrated by expression of autophagy-related factors (LC3-I/II, PINK, PARKIN, and Ub), which are all required to form autophagosomes. Meanwhile, we found the lysosomal acidification (an increased number of lysosomes which had a lower pH value) was increased. Because the formation of autolysosomes primarily depends on the acidification of lysosomes, an increase in lysosomal acidification indicates an active autophagic flux. However, it is worth noting that the P62 protein, serves as a bridge to connect LC3B to the ubiquitylated substrate when forming autophagosomes, was significantly upregulated at both the cellular and mitochondrial levels [51, 52]. This is because the P62 is degraded once autophagosomes fuse with lysosomes [53–56]. Combined with the increased PA-induced autophagy, especially the increased level of lysosomal acidification, the obvious accumulation of P62 protein seemed to be contradictory to expectation. Although an increase or decrease in the levels of P62 protein and aggregates can reflect a change in autophagic activity, P62 expression can also be regulated at the transcriptional level [57]. For instance, exercise or starvation, up-regulated the mRNA levels of *P62* in muscles [58, 59]. Particularly our data showed the mRNA expression of *P62* following PA treatment was several-fold higher than that of the control group, which might mask autophagy induced P62 degradation despite an increase in autophagic flux. Besides, P62 is degraded slowly as an autophagy substrate [60], and our PA treatment was only 12 h, thus the upregulation of P62 may relate to the reduced time available for degradation. Combined with the high rate of mitochondrial abnormity in the PA group, we hypothesize that PA could activate mitochondrial autophagy, at least to a certain extent; however, the efficacy of PA was not sufficient to effectively remove ruptured mitochondria in the BEECs.

MT not only acts as an antioxidant, but it is also considered as a potential drug to intervene obesity based on a positive effect on regulating metabolism [61, 62]. We found BEECs, subjected to a 1 h incubation with 100 μmol/L MT prior to PA, reversed the PA-induced mitochondrial oxidant stress and dysfunction, and further enhanced the functioning of the mitochondrial respiratory chain and diminished apoptosis. Similarly, the treatment of HepG2 cells with 1 mmol/L MT for 30 min before PA treatment prevented lipid deposition and opening of the mitochondrial permeability transition pore [15]. Moreover, both Crespo et al. and Vânia Brazão et al. [63–65] showed Pre-treated MT, demonstrated by increasing the functional activity of antioxidant enzymes (SOD, glutathione reductase and glutathione peroxidase), prematurely activated the antioxidant defense system in cells to combat against subsequent stimuli. This may explain why our pre-treatment could significantly protect BEECs from PA stress. More than just being an excellent antioxidant, MT has recently been identified to regulate autophagy. Chen et al. [66] found MT could improve cognitive function in patients with Alzheimer disease by improving mitochondrial-lysosomal fusion and by restoring mitochondrial autophagy. Tao et al. [67] also discovered MT promoted autophagy in the peripheral blood mononuclear cells of sheep and reduced the excessive inflammatory response induced by LPS.

Similarly, we found that pre-treatment with MT further enhanced autophagy. It is possible that MT pretreatment actively defends against PA-induced mitochondrial damage through the autophagic system. Mechanistically, AMPK, which is usually activated to increase autophagy during cell starvation treatment to provides energy for cells, was also phosphorylated when the cells were surrounded by PA as an energy substrate [68]. Consistent with previous studies [69, 70], under such high-concentrated PA, it is not one kind of energy substrate, but a source of harmful high-fat stress that could initiate autophagy via the AMPK-dependent signaling pathway to reduce oxidative stress. In addition, the AMPK pathway activates autophagy by phosphorylating at S317 and S777 to inhibit mTOR [71]. Emerging evidence shows that MT could regulate mitochondrial autophagy and protect cells from oxidative stress-induced damage by activating the AMPK-mTOR pathway [72, 73]. As expected, in contrast to the expression levels of p-AMPK, MT decreased the p-mTOR (Ser2448)/mTOR expression levels compared to PA group. Here, we also provided an important clue that the expression levels of Beclin-1 increased after MT pretreatment, indicating further autophagy promoted by MT. Collectively, these results suggested that MT activated the upstream pathways of autophagy, AMPK-mTOR-Beclin-1, to parcel more damaged mitochondria by phagophore [74, 75]. In addition, MT decreased expression levels of LC3B and P62 proteins when compared with PA group, which indicated that MT actively eliminated autophagosomes and enhanced autophagy flux. Therefore, the addition of MT before PA stress may degrade more damaged mitochondria through AMPK-mTOR-Beclin-1 pathway.

Apart from promoting autophagy, Xu et al. [76] and Shen et al. [77] proposed that MT could inhibit autophagy, contributing to cell survival under conditions of oxidative stress. Thus, there is a limitation to our current work in that there is some uncertainty relating to MT-induced autophagy level in all stimulus because the upregulation and downregulation of autophagy by MT is determined by the types and duration of stimulating drugs. Interestingly, the specific function of the increase P62 protein induced by an excess PA is uncertain, which is worth to excavate its behind mechanism. Also, we found there was an increased mRNA and protein expression of BCL2, which can bind Beclin-1 to Class III phosphatidylinositol 3-kinase (PI3KIII), thereby inhibiting the formation of autophagosomes and leading to impaired autophagy [78, 79]. However, Ke et al. [80] reported that the phosphorylation of BCL2 at Ser70 site would promote autophagy, which provides a new idea for our following researches. Another further research interest is to perform in-vivo experiment to explore the specific effect of NEB on embryo implantation. Hence, our findings elucidate the mechanism responsible for how PA causes damage to BEECs, and support a dual role of MT in that it could reduce mitochondrial oxidative stress and remove more damaged mitochondria by increasing autophagy, thereby attenuating the damage caused to BEECs by PA, which provides a mechanism reference for the prevention of reproductive disorders caused by NEB.

## Conclusion

**Fig. 7:**
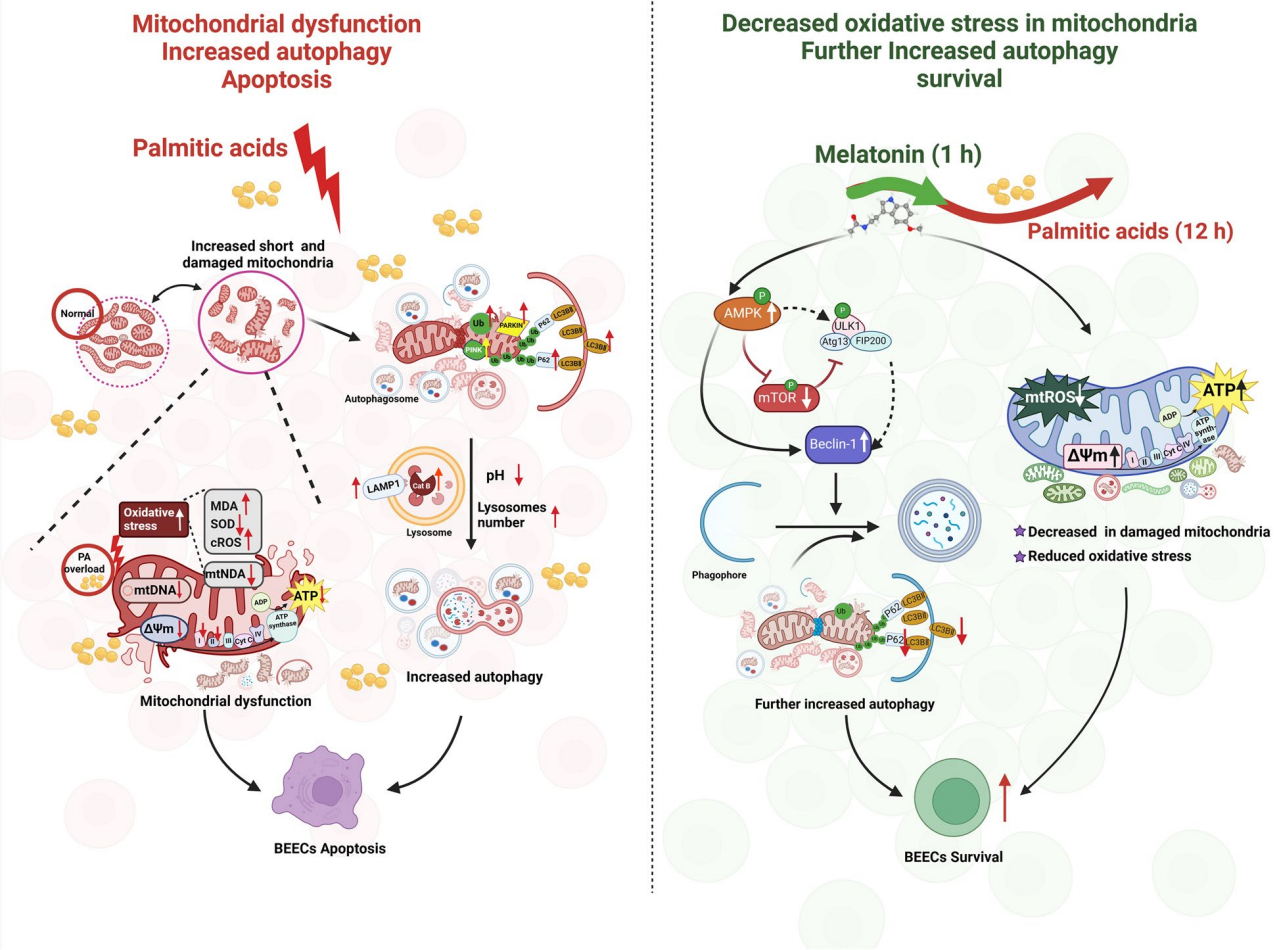
The effect of MT against PA-induced mitochondrial dysfunction and cell injuries in BEECs and the potential mechanism responsible for these effects. The protective effect of MT against PA-induced injury in BEECs and mitochondrial dysfunction may be partly attributed to its ability to enhance autophagy and attenuate mitochondrial oxidative stress

In conclusion, the stimulation of overloaded PA caused mitochondrial oxidative stress and dysfunction in BEECs, leading to apoptosis. Additionally, PA triggered autophagy in BEECs. We revealed that pre-treatment with MT reduced mitochondrial oxidative stress and further enhanced autophagic clearance of damaged mitochondria via the AMPK-mTOR-Beclin-1 pathway, which together alleviated PA-induced mitochondrial dysfunction and BEECs apoptosis (Fig. 7). Our study elucidates the mechanism responsible for how PA causes damage to BEECs and how MT alleviates PA-induced damage in BEECs, which provides a mechanism reference for the prevention of reproductive disorders caused by NEB.

## Data Availability

The datasets used and/or analysed during the current study are available from the corresponding author on reasonable request.

## Abbreviations

BEECs: Bovine endometrial epithelial cells
cROS: Cytosolic reactive oxygen species
ETC: Electron transport chain
MDA: Malondialdehyde
mitoROS: Mitochondrial reactive oxygen species
mtDNA: Mitochondrial copy number
MMP: Mitochondrial membrane potential
MT: Melatonin
NEB: Negative energy balance
NEFA: Non-esterified fatty acid
PA: Palmitic acid
qRT-PCR: Quantitative real-time polymerase chain reaction
ROS: Reactive oxygen species
SDS-PAGE: SDS-polyacrylamide gel electrophoresis
SOD: Superoxide dismutase
TEM: Transmission electron microscopy
